# Evolution of molecular switches for regulation of transgene expression by clinically licensed gluconate

**DOI:** 10.1093/nar/gkad600

**Published:** 2023-07-27

**Authors:** Ana Palma Teixeira, Shuai Xue, Jinbo Huang, Martin Fussenegger

**Affiliations:** Department of Biosystems Science and Engineering, ETH Zurich, Mattenstrasse 26, CH-4058Basel, Switzerland; Department of Biosystems Science and Engineering, ETH Zurich, Mattenstrasse 26, CH-4058Basel, Switzerland; Department of Biosystems Science and Engineering, ETH Zurich, Mattenstrasse 26, CH-4058Basel, Switzerland; Department of Biosystems Science and Engineering, ETH Zurich, Mattenstrasse 26, CH-4058Basel, Switzerland; Faculty of Science, University of Basel, Mattenstrasse 26, CH-4058Basel, Switzerland

## Abstract

Synthetic biology holds great promise to improve the safety and efficacy of future gene and engineered cell therapies by providing new means of endogenous or exogenous control of the embedded therapeutic programs. Here, we focused on gluconate as a clinically licensed small-molecule inducer and engineered gluconate-sensitive molecular switches to regulate transgene expression in human cell cultures and in mice. Several switch designs were assembled based on the gluconate-responsive transcriptional repressor GntR from *Escherichia coli*. Initially we assembled OFF- and ON-type switches by rewiring the native gluconate-dependent binding of GntR to target DNA sequences in mammalian cells. Then, we utilized the ability of GntR to dimerize in the presence of gluconate to activate gene expression from a split transcriptional activator. By means of random mutagenesis of GntR combined with phenotypic screening, we identified variants that significantly enhanced the functionality of the genetic devices, enabling the construction of robust two-input logic gates. We also demonstrated the potential utility of the synthetic switch in two *in vivo* settings, one employing implantation of alginate-encapsulated engineered cells and the other involving modification of host cells by DNA delivery. Then, as proof-of-concept, the gluconate-actuated genetic switch was connected to insulin secretion, and the components encoding gluconate-induced insulin production were introduced into type-1 diabetic mice as naked DNA via hydrodynamic tail vein injection. Normoglycemia was restored, thereby showcasing the suitability of oral gluconate to regulate *in situ* production of a therapeutic protein.

## Graphical Abstract

**Graphical Abstract:**
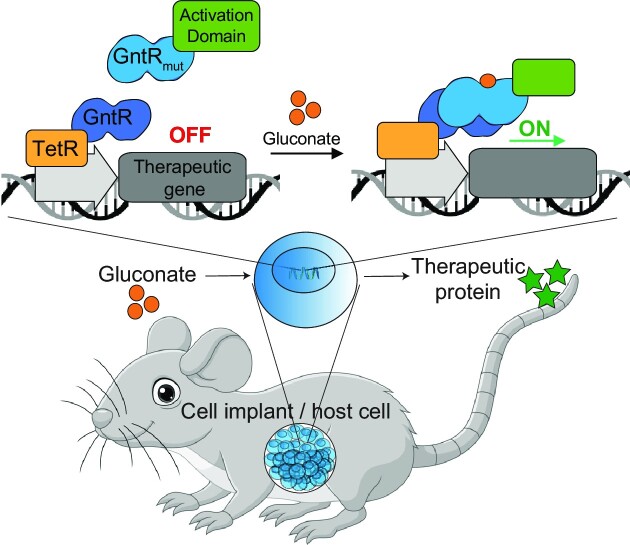

## INTRODUCTION

Synthetic biology can help unlock the potential of gene and cell-based therapies to provide more efficient and safer treatment options for a wide range of chronic diseases, enabling customizable treatment regimens to fit each patient's needs. Despite the ever-expanding pipeline of viral and non-viral gene therapies in clinical trials, this therapeutic modality has not yet achieved widespread clinical success. So far, most research efforts have been directed to vector design in order to improve cell and tissue targeting (1), and less attention has been given to the molecular components that regulate the expression of the therapeutic payloads (2), which are often controlled by constitutive or tissue-specific promoters (3,4). Incorporating synthetic gene-regulatory networks controlled by external signals to achieve precision control over the therapeutic outputs can be a game-changer in the clinical success of gene therapies. Their use can also boost the outcome of other therapies, for example by providing ways to manage over-activation or uncontrolled proliferation of chimeric antigen receptor (CAR)-modified T cells after infusion into patients for inducible cancer immunotherapy (5,6) or by limiting the time window of CRISPR/Cas9 expression to reduce off-target effects in CRISPR/Cas9-mediated *in vivo* gene editing (7).

Several mammalian synthetic gene switches have been designed to enable exogenous control over the magnitude and timing of therapeutic gene expression via specific input signals, ranging from various chemicals, such as antibiotics (8), cancer drugs (9), antivirals (10,11) and food components/additives (12–14), to traceless physical stimuli, such as light (15,16), temperature change (17,18), or electrical signals (19,20). However, the number of mammalian gene switches sensitive to small molecules that are suitable for *in vivo* applications still falls short, as most of the available trigger compounds suffer from pharmacokinetic challenges or undesirable side effects (21). Furthermore, despite the benefits of remote actuation for *in vivo* applications, using physical triggers has some disadvantages, such as the limited depth to which they can penetrate tissue to stimulate modified host cells or implanted engineered cells, or the need for complex equipment to apply the stimulus (21).

Here, we focused on gluconate as a small-molecule inducer that is already clinically licensed, and set out to assemble gluconate-responsive gene switches suitable for both *in vitro* and *in vivo* applications. Gluconate is naturally found in small amounts in foods such as fruits, honey, wine or rice, and is also used in the food industry as an additive in many processed foods, featuring in the list of ingredients generally recognized as safe (GRAS) (22). Furthermore, gluconate is often used as a neutral carrier of ions, such as calcium, zinc or iron, in medications or nutritional supplements used to treat conditions such as anemia and hypocalcemia, or in combination with other drugs such as the anti-parasitic medication sodium stibogluconate used to treat leishmaniasis (23). Although little is known about the metabolism of gluconate in human cells, it is likely converted into the pentose phosphate pathway intermediate 6-phosphogluconate by gluconokinase, as expression of this enzyme has been detected in several human tissues (24) and cell cultures (25,26). This feature would provide better control over the timing/reversibility of transgene expression by preventing prolonged exposure to the inducer.

Some bacteria are capable of using gluconate as a source of carbon and energy in a glucose-restrictive environment. The gluconate catabolic pathway of *E. coli* is controlled by the transcriptional repressor GntR, which contains a DNA-binding helix-turn-helix domain at the N-terminus and an effector-binding domain (EBD) at the C-terminus (27). In *E. coli*, the regulatory regions of gluconate-inducible metabolic genes contain one or two copies of a highly conserved DNA sequence to which GntR binds (28). The affinity of GntR for these sites is impaired in the presence of gluconate due to a conformational change in the gluconate-bound form of GntR, resulting in transcriptional activation.

In this work, we leveraged the ability of gluconate to induce changes in the GntR protein to assemble high-performance gene switches responsive to gluconate in mammalian cells. We applied a combination of random mutagenesis and high-throughput phenotypic screening to identify GntR variants with enhanced gluconate-inducible dimerization of GntR-fused split DNA-binding and transactivation domains, thereby obtaining a greater fold-activation of gene expression. Importantly, we demonstrated that the optimized molecular components could be used to assemble robust Boolean logic gates in cell culture and also to enable oral gluconate-mediated control of transgene expression in wild-type mice. As proof-of-concept, the gene switch was applied in the context of diabetes, in which the orthogonality of the calorie-free inducer could be explored to regulate insulin expression and help managing blood glucose levels within the physiological range. We showed that orally administered gluconate, by itself, has no effect on the fasting or post-prandial glucose levels of type-1 diabetic (T1D) mice, and confirmed that introduction of the components encoding gluconate-switched insulin production restored normoglycemia in T1D mice fed with gluconate.

## MATERIALS AND METHODS

### Plasmid construction

Design and cloning details for all genetic components utilized in this study are provided in Supplementary Tables S1 and S2 (Supporting Information). Plasmids were designed using Benchling (www.benchling.com) and constructed by standard molecular cloning techniques. For restriction enzyme-based cloning, plasmids were digested with the desired endonucleases (New England Biolabs); digested backbones were dephosphorylated with Quick CIP (M0525L, New England Biolabs) before ligation with T4 DNA ligase (EL0011, Thermo Fisher). All PCR reactions were performed using Q5 High-Fidelity DNA polymerase (M0491L, New England Biolabs). After ligation, the plasmids were transformed and amplified in *E. coli* XL10-Gold strain (Agilent) and DNA was extracted using a plasmid miniprep kit (Zymo Research) or Midiprep Kit (D4200, Zymo Research). Constructs were verified by Sanger sequencing at Microsynth AG. Synthetic gene fragments used in the study were codon-optimized for expression in human cells and synthesized by Twist Bioscience.

### Cell culture

Human embryonic kidney cells (HEK-293T, ATCC: CRL-11268) were cultivated in Dulbecco's modified Eagle's medium (DMEM; 10566016, Thermo Fischer) supplemented with 10% v/v fetal bovine serum (FBS; F7524, Sigma Aldrich) under a humidified atmosphere containing 5% CO_2_ at 37°C. Routine sub-culture was performed every 2–3 days when cell plates were around 80% confluent. Cells were detached using 0.05% trypsin–EDTA (25300054, Life Technologies) and re-seeded in fresh medium at the desired cell density. Cell number and viability were quantified using a CellDrop automated cell counter (DeNovix).

### Transient transfection

For transfection experiments in 96/48/24-well plates (Corning), cells were seeded at 0.1×10^6^ cell/ml, the day before transfection. The transfection mixture for one well of 96-well plates consisted of 50 μl of FBS-free DMEM containing a 1:4 DNA:PEI (polyethylenimine, MW 40000; 24765, Polysciences) mixture, with a total DNA amount of 120–150 ng. For larger plate formats, the quantities were scaled up accordingly. After 20 min incubation at room temperature, the transfection mixture was added dropwise to the cells, which were then incubated overnight. The next morning, the culture medium was replaced with fresh FBS-containing DMEM with or without sodium gluconate (527-07-1, Merck) and cell supernatant samples were collected 24 h later for assay of human placental secreted alkaline phosphatase (SEAP) or nanoluciferase (NLuc) reporter, unless otherwise indicated.

### Generation of monoclonal stable cell lines

HEK-293T cells were transfected with a hyperactive Sleeping Beauty (SB) transposase (pTS395) expression vector (29) in a 1:5 (weight/weight) ratio with pAna366 and pAna368 vectors, containing SB recognition sites and encoding a puromycin resistance marker and a blue fluorescent protein. The medium was exchanged 12 h after transfection and cells were incubated for 48 h before the addition of selection medium containing 4 μg/mL puromycin. After three passages, the polyclonal population was sorted by fluorescence-activated cell sorting into single cells in 96-well plates and expanded to obtain monoclonal cell lines.

### Random mutagenesis of GntR

The library of GntR mutants was synthesized using a GeneMorph II random mutagenesis kit (200552, Agilent Technologies) according to the manufacturer's instructions. A PCR reaction was performed to amplify the GntR sequence in the GntR-VPR fusion protein (pAna274), and the product was then cloned in the template plasmid, replacing the wild-type GntR. The library was transformed into competent XL10-Gold *E. coli*, plated into ampicillin-containing agar plates and incubated overnight at 37 °C. Colonies were picked and expanded during 24 h in deep 96-well plates containing 750 μl of ampicillin-supplemented LB medium, at 37 ^o^C with orbital shaking (220 rpm). The plasmid library was purified using the Zyppy-96 plasmid mini prep kit (D4041, Zymo Research) according to the manufacturer's instructions.

### SEAP quantification

Heat-inactivated (30 min, 65°C) cell culture supernatants (20 μl) were transferred into each well of a 96-well plate (260836, Thermo Fisher) and mixed with 80 μl water, 80 μl 2 × SEAP buffer (20 mm homoarginine, 1 mm MgCl_2_, 21% v/v diethanolamine, pH 9.8), and 20 μl substrate solution containing 20 mm*para*-nitrophenyl phosphate (*p*NPP; 128860100, Acros Organics BVBA). The absorbance of samples was measured at 405 nm using a Tecan Infinite M1000 plate reader (Tecan AG) at 37°C.

### NLuc quantification

Secreted NLuc in cell culture supernatants was measured with the Nano-Glo Luciferase Assay System (N1110, Promega). In brief, a 7.5 μl aliquot of each sample was mixed with 7.5 μl buffer/substrate mix (50:1) in the wells of 384-well plates (781076, Greiner Bio One), which were then incubated at room temperature for 10 min. Luminescence was measured with a Tecan M1000 plate reader.

### Insulin quantification

Recombinant mINS levels in serum were quantified using a mouse insulin ELISA kit according to the manufacturer's instructions (10-1247-01, Mercordia). Optical density was measured at 450 nm on a Tecan M1000 plate reader and the corresponding concentrations were calculated in Prism 9 (GraphPad Software Inc) using a cubic-spline regression based on the measured absorbances of manufacturer-provided standard solutions.

### Animal experiments

C57BL/6JRI mice (Janvier Labs Saint-Berthevin, France), weighing ∼25 g, were used. Animals were housed in a controlled room at 22°C, 50% humidity, 12-h light-dark cycle with ad-libitum access to standard diet and drinking water. Animals were randomly assigned to experimental groups. To induce type-1-diabetes, C57BL/6JRI mice (male, 8–9 weeks) were treated during 5 days with streptozocin (S0130, Merck) at a daily dose of 75 mg/kg to deplete insulin-producing beta cells. Liver function was evaluated by assaying the serum levels of alanine transaminase (ALT, ab105134, Abcam) and aspartate aminotransferase (AST, MAK055, Sigma Aldrich) according to the manufacturers’ protocols. The body weight of the animals was also regularly monitored. *Cell implants*. Engineered HEK-293T cells were encapsulated into coherent alginate-poly-(l-lysine)-alginate beads (400 μm; 200 cells/capsule) using an Inotech Encapsulator Research Unit IE-50R (Buechi Labortechnik AG) with the following parameters: 20 ml syringe operated at a flow rate of 400 units, 200 μm nozzle with a vibration frequency of 1200 Hz, and bead dispersion voltage of 1.5 kV. Wild-type mice were intraperitoneally injected with 1 ml of DMEM containing 5×10^6^ cells. Sodium gluconate solutions were supplemented by oral gavage (250 μl). Thai rice was pulverized, mixed with water (ratio 1:1) and administered to mice by o.g. at a dosage of 5 g/kg (equivalent to 375 g for a human weighing 75 kg). *Hydrodynamic injection*. Mice were injected with 2 ml of saline solution containing the plasmids pAna366 and pAna368 (4 mg/kg of DNA per mouse) 24 h before starting inducer treatment. Gluconate administration was performed twice/day (e.g. 5 g/kg). Fasting blood glucose was measured after 4 h of food restriction using a clinically licensed glucometer (Accu-Check Instant, Roche). Glucose tolerance test (GTT) was performed by i.p. injection of glucose (2 g/kg), and glycemia was profiled at intervals for the following 2 h. Serum was isolated for analysis using BD Microtainer® SST tubes according to the manufacturer's instructions (30 min incubation in the dark, centrifugation for 2 min at 10 000 × g; 365967, Becton Dickinson). All experiments involving animals were performed in accordance with the Swiss animal welfare legislation, approved by the veterinary office of the Canton Basel-Stadt (approval no. 2879/31996) and carried out by Shuai Xue (no. LTK4899) and according to the directives of the European Community Council (2010/63/EU), approved by the French Republic (project no. DR2018-40v5 and APAFIS #16753), and carried out by Shuai Xue and Ghislaine Charpin-El Hamri (no. 69266309) at the University of Lyon, Institut Universitaire de Technologie (IUT), F69622 Villeurbanne Cedex, France.

### Statistical analysis

Statistical evaluation was conducted by using an unpaired Student's two-tailed *t*-test for comparing two sets of data as implemented in Prism GraphPad 9 (GraphPad Software Inc., San Diego, California, USA).

## RESULTS

### Gluconate gene switches based on GntR affinity for specific DNA sequences

**Figure 1.**
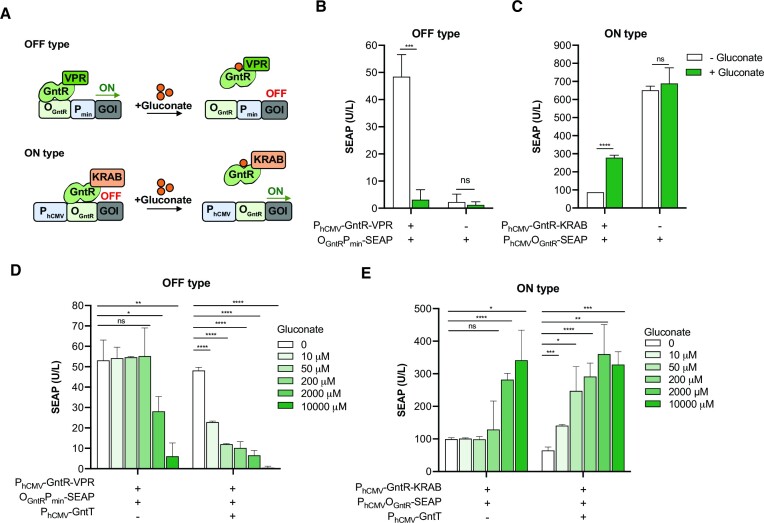
Gluconate gene switches based on GntR binding to its cognate DNA operator site. (**A**) Scheme depicting gluconate-sensitive genetic circuits with OFF- and ON-type behavior in mammalian cells. The OFF system consists of GntR fused to a mammalian transactivator (VPR), which in the absence of gluconate binds to a GntR operator sequence (O_GntR_) placed upstream of a minimal promoter (P_min_), thereby driving expression of the downstream gene. In the presence of gluconate, GntR undergoes a conformational change and can no longer bind to the promoter region, thereby switching off expression. In the switch-ON system, GntR fused to a trans-silencer domain (KRAB) binds to the O_GntR_ sequence placed downstream of a constitutive promoter (P_cons_) in the absence of gluconate, thereby preventing transgene expression. In the presence of gluconate, the synthetic repressor loses affinity for O_GntR_ and transgene expression is switched on. (**B**) SEAP production by transiently transfected HEK-293T cells with constitutive expression of GntR fused to the strong mammalian transactivator VPR (pAna274, P_hCMV_-GntR-VPR-pA) and the reporter plasmid (pAna268, O_GntR_P_min_-SEAP-pA), and cultured for 24 h in the presence or absence of gluconate (10 mM). (**C**) Characterization of the switch-ON system based on GntR fused to a KRAB domain. SEAP expression from cells transfected with pAna283 (P_hCMV_-GntR-KRAB-pA) and pAna299 (P_hCMV_-O_GntR_-SEAP-pA), and cultured for 24 h in the presence or absence of gluconate (10 mM). (**D**) Dose-response relationship of the switch-OFF system in the presence or absence of constitutive expression of the gluconate transporter GntT. SEAP expression from cells transfected with pAna268 (O_GntR_-P_min_-SEAP-pA), pAna274 (P_hCMV_-GntR-VPR-pA), and either pAna302 (P_hCMV_-GntT-pA) or an empty plasmid, and then cultured for 24 h in the presence or absence of different gluconate concentrations. (**E**) Dose-response relationship of the switch-ON system in the presence or absence of constitutive expression of GntT. SEAP expression from cells transfected with pAna268, pAna274, and either pAna302 or an empty plasmid, and then cultured for 24 h in the presence or absence of different gluconate concentrations. Data are shown as mean ± SD of *n* = 3 biologically independent samples, representative of 3 independent experiments. ns, not significant, * *P* < 0.05, ** *P* < 0.01, *** *P* < 0.001, **** *P* < 0.0001.

We sought to build gluconate-regulated gene expression systems with OFF- and ON-type behavior in mammalian cells by capitalizing on the gluconate-mediated GntR interactions with known DNA sequences in *E. coli* (Figure 1A). The OFF-type switch relies on GntR fused to the strong tripartite mammalian transactivator VP64-p65-Rta (VPR), while the ON-type switch relies on GntR fused to the transrepressor Kruppel-associated box (KRAB) domain from human ZNF10. In the absence of gluconate, these fusion proteins bind to GntR-recognition DNA sequences (O_GntR_), thereby activating or repressing, respectively, the transcription of downstream genes. HEK-293T cells transiently transfected with the switch-OFF system controlling the expression of secreted alkaline phosphatase (SEAP) as a reporter protein showed significant gluconate-responsiveness, producing 10-fold less SEAP in the presence of gluconate (Figure 1B). We tested two GntR DNA recognition sites: (i) the sequence found in the promoter region of the gluconate transport operon (ATGTTACCGATAACAG) and (ii) the consensus sequence (ATGTTACCCGTAACAT) (28). The greatest responsiveness to gluconate was obtained with the first sequence (Supplementary Figure S1A). To further assess the specificity of GntR-VPR for the target sequence, we also deleted the whole O_GntR_ operator site from the promoter region or inserted two base pairs in the center of this sequence. Both mutations abolished SEAP expression from the minimal promoter in the presence of gluconate (Supplementary Figure S2). The switch-ON system showed repression of SEAP expression from the constitutive human cytomegalovirus promoter (P_hCMV_) bearing downstream GntR-binding sequences when the cells were cultured in gluconate-free medium and GntR-KRAB was constitutively co-expressed (Figure 1C). In the presence of gluconate, SEAP production was significantly increased. Two or three tandem repeats of the binding site in the promoter region did not improve the performance of the switches (Supplementary Figure S1A, B), and neither did replacing the constitutive promoter P_hCMV_ with the phosphoglycerate kinase promoter (P_PGK_) in the ON-type switch (Supplementary Figure S1C). We also confirmed that each domain (GntR, VPR, KRAB) in the OFF- and ON-type switches is essential to obtain gluconate-mediated gene expression (Supplementary Figure S3A, B). The responsiveness is improved when the O_GntR_ sequence is placed upstream of the TATA box in the OFF-type switch (rather than downstream), and downstream of the constitutive promoter (P_hCMV_) in the ON-type switch (rather than upstream) (Supplementary Figure S4A, B). Next, we tested whether co-expression of the high-affinity gluconate transporter *gntT* from *E. coli* (28) would increase the sensitivity of the gene switches. Engineered cells cultured in the presence of different gluconate concentrations showed significant fold-change differences of SEAP expression at concentrations as low as 10 μM for both the ON- and OFF-type systems when co-expressing GntT (Figure 1D, E).

### Gluconate-inducible gene expression relying on GntR dimerization

**Figure 2.**
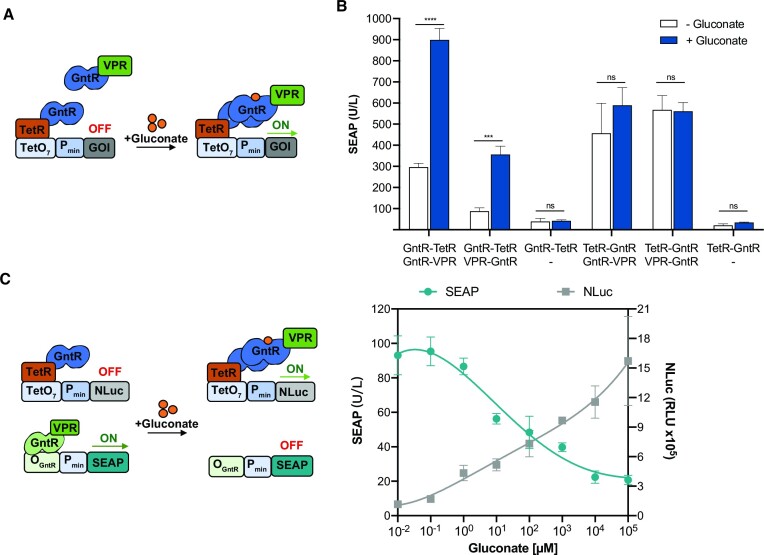
Switch-ON system relying on gluconate-induced GntR dimerization. (**A**) Switch design. Dimerization of the two separate fusion proteins GntR-TetR and GntR-VPR in the presence of gluconate colocalizes VPR near a promoter region containing tetracycline-responsive elements (TetO_7_-P_min_), allowing expression of the downstream gene. (**B**) SEAP expression from cells transfected with pTS1017 (TetO_7_-P_min_-SEAP-pA), with constitutive expression of GntR fused to TetR (P_hCMV_-GntR-TetR-pA or P_hCMV_-TetR-GntR-pA) and either constitutive expression of GntR fused to VPR (P_hCMV_-GntR-VPR-pA or P_hCMV_-VPR-GntR-pA) or an empty vector, and then cultured for 24 h in the presence or absence of gluconate (10 mM). (**C**) Controlling the expression of two reporter proteins (SEAP and NLuc) in response to gluconate. Genetic constructs (left) and reporter analysis (right). HEK-293T cells were transiently transfected with the switch-ON system based on the TetR-GntR interaction with a TetO_7_ DNA sequence driving NLuc expression and the switch-OFF system based on the GntR-VPR interaction with an O_GntR_ DNA sequence driving SEAP expression. Supernatant levels of NLuc and SEAP were analyzed 24 h after incubation in the presence of different gluconate concentrations. Data are shown as mean ± SD of *n* = 3 biologically independent samples, representative of three independent experiments. ns, not significant, *** *P* < 0.001, **** *P* < 0.0001.

We have recently shown that some bacterial transcription factors (TFs) comprising helix-turn-helix (HTH) DNA-binding domains linked to an effector-binding domain (EBD) also show responsiveness to their effectors when fused to another TF, relying on the interaction of the latter TF with its cognate DNA-binding sequence for the regulation of gene expression (30). To test whether this approach would also work for GntR, we transfected HEK-293T cells with constitutively expressed GntR fused to either TetR or to the VPR transactivator and analyzed gluconate-inducible SEAP expression from a tetracycline-responsive promoter. The underlying hypothesis is that GntR dimerization brings VPR near the target promoter, thereby activating SEAP expression in a gluconate-responsive manner (Figure 2A). We tried fusions of TetR and VPR at both termini of GntR and observed gluconate-inducible SEAP expression in cells transfected with GntR-TetR incorporating VPR at the N- or C-terminus of GntR (Figure 2B), affording a 3- or 4-fold increase in SEAP expression, respectively. In a cytosolic dimerization assay based on split nanoluciferase (NLuc), in which the N- and C-parts of NLuc are fused to either terminus of GntR, we observed an increased NLuc signal in the presence of gluconate for three out of four combinations. The highest fold-induction was observed when both NLuc split parts were fused to the C-terminus of GntR (Supplementary Figure S5), and therefore we adopted the GntR-VPR/TetR fusion configurations in all subsequent experiments. To regulate the expression of two proteins simultaneously, we combined this ON-type gene switch with the OFF-type gene switch based on a synthetic promoter bearing gluconate-responsive elements (Figure 1B). Increasing concentrations of gluconate resulted in increased secretion of NLuc and decreased secretion of SEAP (Figure 2C). In this scenario, the synthetic transcription factor GntR-VPR functions simultaneously to control two opposite expression states: (i) in low gluconate-containing medium, SEAP expression is ON and NLuc expression is OFF, while in high gluconate-containing medium, SEAP expression is OFF and NLuc expression is ON.

### Optimizing gene switches via random mutagenesis of GntR and screening

**Figure 3.**
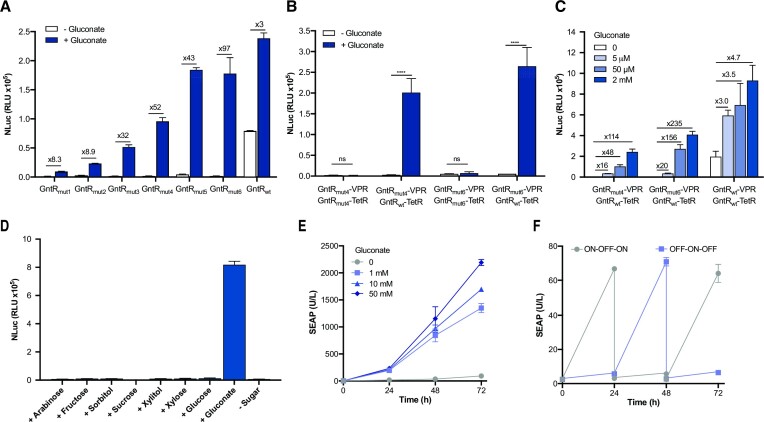
Optimization of a gluconate-sensitive genetic circuit. (**A**) Secondary screening of GntR variants obtained by error-prone PCR, coupled with a high-throughput functional assay based on NLuc secretion in 96-well plate cultures. Nluc secretion levels from HEK-293T cells transfected with plasmids encoding six different GntR_mut_-VPR variants, along with GntR_wt_-TetR and TetO_7_-P_min_-NLuc, and exposed or not exposed to gluconate (5 mM) for 24 h. (**B**) Effect of using the same GntR mutant (GntR_mut4_ or GntR_mut6_) in both VPR and TetR fusion constructs. NLuc secretion levels from HEK cells transfected with homodimerizing (GntR_mut_-VPR/GntR_mut_-TetR) or heterodimerizing (GntR_mut_-VPR/GntR_wt_-TetR) partners, and incubated with or without gluconate (10 mM). (**C**) Dose–response relationship of gene switches using different GntR variants (GntR_mut4_, GntR_mut6_ and GntR_wt_) in the presence of constitutive expression of GntT. NLuc expression from transfected cells cultured for 24 h in the presence or absence of different gluconate concentrations. (**D**) Specificity for gluconate. Other sugars and structurally similar molecules failed to trigger significant NLuc expression. (**E**) Expression kinetics. HEK-293T cells genetically engineered with gluconate-responsive SEAP production were cultured for 72 h in different gluconate concentrations, and sampled daily for SEAP analysis. (**F**) Reversibility performance. HEK-293T cells genetically engineered with gluconate-responsive SEAP production were cultivated for 72 h, with daily medium exchange, alternating between gluconate-containing (1 mM) or gluconate-free medium. Data are shown as mean ± SD of *n* = 3 biologically independent samples, representative of three independent experiments. Numbers above the bars indicate fold difference in NLuc expression level between gluconate-treated and non-treated cultures. ns, not significant, **** *P* < 0.0001.

We sought to improve the genetic circuit output by generating error-prone PCR-based random mutations of the GntR sequence in the GntR-VPR fusion protein. We evaluated the performance of the GntR-VPR mutant library by means of a high-throughput functional screening assay, and selected the best candidates in terms of sensitivity and fold-induction of NLuc expression from a tetracycline-responsive promoter (Supplementary Figure S6A). HEK-293T cells co-transfected with the wild-type GntR-TetR and the library of GntR-VPR variants were assayed for NLuc secretion after incubation for 24 h in the presence or absence of gluconate (Supplementary Figure S6B). Six hits were validated in a secondary screening, and confirmed to provide significantly higher fold-inductions than the wild-type GntR-VPR fusion protein (Figure 3A), due mostly to significant lower basal NLuc expression in the absence of gluconate. Interestingly, the three best performing mutants (mut4 GntR_D55V/E235K/G279V_, mut5 GntR_A306S_, and mut6 GntR_R285C/V289I_), have a single mutation or double mutations on the C-terminal EBD, spanning between residues 161 and 331 (31), and all of them involved replacement of at least one polar amino acid residue by a nonpolar residue, or vice versa (Supplementary Table S3). These changes might favor dimerization with the wild-type GntR fused to TetR, resulting in increased activation of NLuc expression.

To compare the gluconate-mediated inducibility of GntR heterodimers with GntR homodimers, we cloned the two GntR mutants that afforded the highest fold-inductions (GntR_mut4_ and GntR_mut6_) in the TetR construct. Interestingly, the inducibility was completely abolished when both dimerizing partners were based on the same GntR variant (Figure 3B). In the cytosolic dimerization assay, while GntR_mut6_ and GntR_wt_ fused to each NLuc moiety increased the NLuc luminescence in the presence of gluconate, use of the GntR_mut6_ variant in both split Nluc moieties did not result in increased luminescence in the presence of gluconate (Supplementary Figure S7), suggesting that gluconate-inducible GntR homodimerization is less favorable for this mutant.

We characterized the dose-response relationships of the two best mutants, GntR_mut4_ and GntR_mut6_, when they were co-expressed with the gluconate transporter GntT (Figure 3C). Both mutants afforded a high dynamic range, with up to 235-fold induction in NLuc levels for 2 mM gluconate and 20-fold in the low micromolar range. We also tested the specificity of the GntR_mut6_-based gene switch for gluconate by evaluating whether a set of different sugars and structurally similar molecules could activate reporter gene expression. None of the screened compounds triggered significant NLuc expression (Figure 3D), suggesting high selectivity of the developed gene switch for the sugar acid gluconate. Next, we assessed how the genetic device responds to gluconate when stably integrated in the cell genome using the Sleeping Beauty transposase system. During this process we replaced VPR with the shorter transactivation domain VP16, which provided comparable functionality of the gene switch (Supplementary Figure S8). SEAP production by transgenic cells treated with gluconate increased dose-dependently and accumulated gradually in the supernatant during 72 h post-induction (Figure 3E). Lastly, we confirmed the reversibility of the gene switch by daily switching between gluconate-containing and gluconate-free medium, during three days to obtain robust ON-OFF-ON expression profiles (Figure 3F).

Altogether, random mutagenesis combined with functional phenotype screening enabled the identification of GntR variants that support high transgene expression levels in the presence of gluconate while maintaining minimal expression in the absence of gluconate, for both transiently provided or stably integrated gene switches.

### Construction of 2-input logic gates

**Figure 4.**
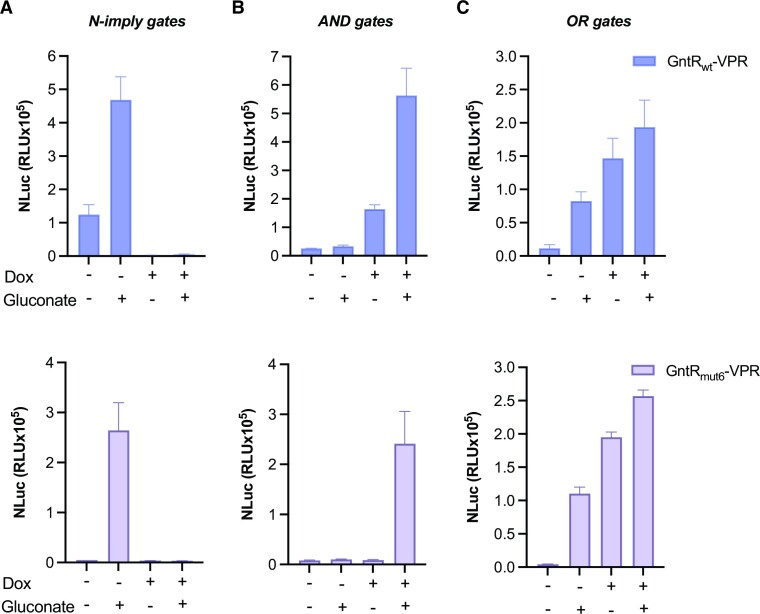
Two-input logic gates. (**A**) N-imply gates. NLuc secretion by cells constitutively expressing GntR_wt_/GntR_mut6_-VPR and GntR-TetR, and NLuc downstream of a tetracycline-responsive promoter. (**B**) AND gates. NLuc secretion by engineered cells constitutively expressing GntR_wt_/GntR_mut6_-VPR and GntR-rTetR, and NLuc downstream of a tetracycline-responsive promoter. (**C**) OR gates. NLuc secretion by engineered cells constitutively expressing GntR_wt_/GntR_mut6_-VPR, GntR-TetR and rTetR-VP16, and NLuc downstream of a tetracycline-responsive promoter. (A–C) The transfected cells were cultured for 24 h in the presence or absence of gluconate (10 mM) or Dox (1 μg/ml), as indicated. Data are shown as mean ± SD of *n* = 3 biologically independent samples, representative of three independent experiments.

The reliance of the gene switches on TetR binding to its cognate DNA sequence and gluconate-inducible dimerization of GntR enabled us to build compact 2-input (gluconate and doxycycline (Dox)) logic gates to regulate the expression of one output. Therefore, we next assessed the performance of logic gates based on GntR_wt_ and GntR_mut6_ in the presence or absence of gluconate and Dox. The N-imply gate (A AND NOT B, in which A is gluconate and B is Dox) using GntR_mut6_ performed better than the gate using GntR_wt_, with average ON/OFF ratios of 64 and 11, respectively. As expected, the addition of Dox significantly decreased reporter gene expression in the presence or absence of gluconate for both GntR variant-based gates (Figure 4A). We also assessed how this gate performs when stably integrated in the cell genome, and confirmed that the addition of Dox overrides the gluconate inducibility (Supplementary Figure S9), thereby constituting a ‘safety-switch’ in a gene/cell therapy setting to stop expression if required. Then, we replaced TetR with reverse TetR (rTetR) to generate logic gates that perform AND operation, in which the presence of both inducers is required to turn on gene expression. The circuit based on GntR_mut6_ performed robustly, showing an average of 27-fold induction between the ON and OFF states (Figure 4B). To assemble gene switches that function as 2-input OR gates, i.e. the output is ON when either or both inputs are present, the three fusion proteins GntR_mut6_-VPR, GntR_wt_-TetR and rTetR-VP16 were constitutively expressed to regulate NLuc expression. The gates functioned as expected, turning NLuc expression ON in the presence of at least one of the inputs (Figure 4C). Although the output signals were higher in the presence of both inducers, the average ON/OFF ratios were high, especially for the gate based on the GntR_mut6_ variant (44-fold vs 12-fold for the GntR_wt_ variant).

### *In vivo* regulation of transgene expression

**Figure 5.**
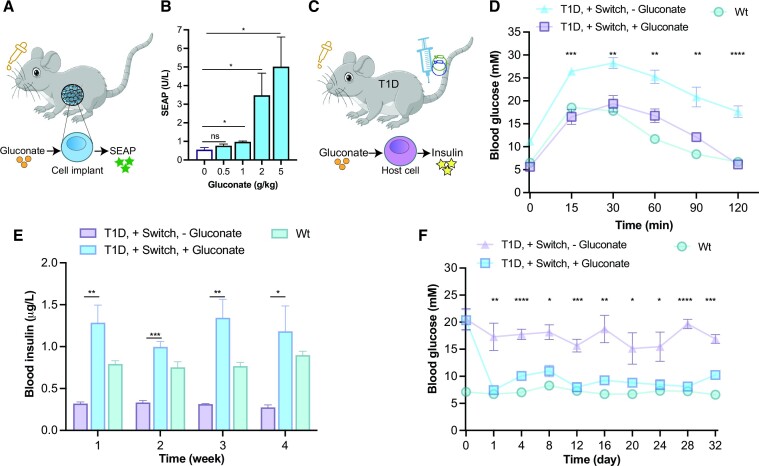
Validation of the gluconate-inducible gene circuit to regulate transgene expression in mice. (**A**) Cells engineered for gluconate-regulated transgene expression and encapsulated in immune-protective alginate beads were intraperitoneally implanted in mice. (**B**) Dose-dependent control of transgene expression. Blood SEAP levels of wild-type mice administered with aqueous solutions of different concentrations of sodium gluconate by oral gavage (o.g.), 2 days after cell implantation. (**C**) Plasmid DNA encoding gluconate-regulated insulin secretion delivered to type-1-diabetic mice via hydrodynamic injection. (**D**) Glucose tolerance test (GTT). T1D mice received the plasmids pAna366 and pAna368 encoding the switch components, via hydrodynamic injection. The GTT was performed by i.p. administration of d-glucose 3 days after transfection and after fasting for 12 h. (**E**) Fasting blood insulin levels during four weeks of treatment. Mice injected with the gene switch were either left untreated or treated with gluconate twice per day. Wild-type mice were included as controls. (**F**) Fasting glucose levels during four weeks of treatment. Mice injected with the gene switch were either left untreated or treated with gluconate twice per day. Data are shown as mean ± SEM of n = 4 mice. Statistical significance was calculated by unpaired t-test (gluconate-treated versus untreated mice). ns, not significant, *P < 0.05, **P < 0.01, ***P< 0.001, **** *P* < 0.0001.

To demonstrate the functionality of our optimized gluconate-actuated genetic switch *in vivo*, we encapsulated engineered cells in alginate and implanted them in wild-type mice, which were then orally given different gluconate concentrations (Figure 5A). Analysis of blood samples revealed a gluconate-dependent increase in SEAP levels (Figure 5B). Conversely, cell implants harboring a non-functional gene switch (lacking the GntR-VP16 expression cassette) did not show gluconate-responsiveness (Supplementary Figure S10). Furthermore, to test whether transgene expression could be inadvertently activated by diet, mice transplanted with functional switches were fed a large amount of rice, which is one of the richest gluconate-containing foods (32). SEAP levels were not increased relative to those of mice that did not receive any gluconate source (Supplementary Figure S10). We next sought to assess whether the gluconate-switchable device could be used to control the expression of therapeutically relevant cargos. We selected type-1 diabetes (T1D) as a disease model to showcase the potential of the device for regulating advanced therapies in response to exogenous gluconate. The T1D mice received the switch components encoding gluconate-induced insulin production as naked DNA via hydrodynamic tail vein injection (Figure 5C), which is a well-established technique for efficient DNA uptake by hepatocytes (approximately 40% of the liver cells have been reported to express the exogenous genes (33)), with much lower vector delivery to other tissues, such as the heart, lungs or kidneys (33,34). To confirm successful DNA delivery into host cells and responsiveness to gluconate, we first established that wild-type mice injected with the components encoding gluconate-switched NLuc expression exhibited higher luminescence in the abdominal region when treated with gluconate (Supplementary Figure S11). We also confirmed gluconate-dependent insulin secretion in transiently transfected cell cultures (Supplementary Figure S12). Gluconate intake by the engineered T1D mice rapidly restored normoglycemia during a glucose tolerance test (GTT), while engineered T1D mice that did not take the inducer remained hyperglycemic (Figure 5D). Moreover, gluconate-treated T1D mice showed significantly higher blood insulin levels (Figure 5E) and lower fasting glucose levels compared to the untreated control group (Figure 5F), suggesting that the engineered host cells can produce enough insulin to regulate blood glucose levels. Gluconate intake by engineered T1D mice did not affect body-weight gain or liver function, as assessed in terms of the serum levels of ALT and AST, over the 4-week treatment period (Supplementary Figure S13). To confirm that gluconate by itself does not affect the diabetic state, we measured fasting blood glucose levels and ran GTTs in T1D mice pre-treated with gluconate or vehicle only. The response profiles were very similar in these two mouse groups (Supplementary Figure S14A, B). Collectively, these results indicate that both the DNA-based and cell-based platforms are functional *in vivo* and can be regulated by exogenously supplemented gluconate.

## DISCUSSION

Synthetic molecular switches enabling tight control over gene expression in mammalian cells in response to environmental signals are useful tools to study basic cellular functions of genes of interest, as well as to develop safer and more efficient gene and engineered cell therapies. In this work, we focused on the non-toxic and bioavailable inducer gluconate and we assembled a number of gluconate-dependent transcriptional control devices featuring robust operation both *in vitro* and *in vivo*. Akin to the classical Tet-Off system, GntR fused to a transactivating domain binds to its cognate DNA-binding sequence O_GntR_ and fully activates transcription of the downstream genes in the absence of the inducer. Fusion of GntR to a trans-silencer domain was required to repress expression in mammalian cells cultured in gluconate-free medium. While ON-type switches are preferable for *in vivo* applications, the reliance on inhibition of a full promoter by the ZNF10 KRAB domain is often associated with a high level of leaky expression and slow activation kinetics (35,36). This prompted us to assemble a second switch configuration featuring ON-type behavior, in which gluconate-inducible dimerization of two separate GntR polypeptides fused to the two domains of a split transcription factor (the DNA-targeting domain TetR and a transactivation domain (VPR or VP16)) come together to activate transgene expression in the presence of gluconate. Through random mutagenesis and functional phenotypic screening, we were able to identify pairs of GntR variants that showed minimal signal output in the absence of gluconate, and high activation in the presence of gluconate, greatly surpassing the gene expression fold-changes achieved with the wild-type GntR protein. The best mutant switch can sense and respond to gluconate in the low micromolar range and achieve expression changes of several hundred-fold in the low millimolar range. Thus, randomly mutating GntR and screening a relatively small mutant library for the retention of function in the presence of the effector proved to be an efficient strategy to find variants that enable improved gluconate-mediated transcriptional control. When we challenged the specificity of the interaction with gluconate by using a set of sugar molecules, none of the sugars tested induced transgene expression.

The optimized molecular components could be combined to assemble efficient higher-order gene circuits, including dual-input logic gates, which exhibited selective AND and OR operations conditioned by exposure to the two small molecules gluconate and Dox. These circuits are expected to be useful for applications requiring combinatorial computation in complex cellular environments.

*In vivo* experiments showed that orally available gluconate can stimulate engineered cell implants microencapsulated in alginate beads, as well as engineered host cells, to express the output signal. Administration of gluconate twice a day in T1D mice triggered the release of enough insulin to restore normoglycemia, as well as to rapidly attenuate postprandial glycemic excursions in glucose tolerance tests. The advantages of gluconate as inducer for controlling gene- and engineered cell-based therapies include its excellent safety profile, given its extensive use in many drug formulations (e.g. 37) and as a common additive in many processed foods (38), such as those labeled with E576 (sodium gluconate), E577 (potassium gluconate), E578 (calcium gluconate), or E579 (ferrous gluconate). While we used hydrodynamic transfection for a proof-of-concept gene therapy application, other more clinically suitable delivery vehicles could alternatively be used. For example, the size of the gluconate-inducible insulin secretion device is compatible with the 8.5 kb packaging capacity of third-generation adenoviral vectors (39). Furthermore, for clinical translation as a cell therapy, more clinically relevant cells would need to be considered, such as mesenchymal stem cells or memory B cells (40–42), engineered with the necessary components to produce the therapeutic output in response to gluconate ingestion. While we selected T1D as the disease model and insulin as the output for the proof-of-concept study, the gluconate-actuated genetic switch should be readily adaptable to control *in situ* production and dosing of other therapeutic proteins for the treatment of a wide range of chronic diseases.

## Supplementary Material

gkad600_Supplemental_File

## Data Availability

The authors declare that all the data supporting the findings of this study are available within the paper and its supporting materials. All original plasmids listed in Supporting Table S2 are available upon request.
