# Supplementary material for: Evolution of molecular switches for regulation of transgene expression by clinically licensed gluconate
Source: Nucleic Acids Res. 2023 Jul 27;51(15):e85. doi: 10.1093/nar/gkad600 (PMC10450161; doi:10.1093/nar/gkad600)
Supplement: gkad600_Supplemental_File [file gkad600_supplemental_file.docx]

**Supplementary Information**

**Evolution of molecular switches for regulation of transgene expression by clinically**

**licensed gluconate**

Ana Palma Teixeira^1+^, Shuai Xue^1+^, Jinbo Huang^1^, Martin Fussenegger^1,2*^

^1^Department of Biosystems Science and Engineering, ETH Zurich, Mattenstrasse 26, CH-4058 Basel, Switzerland.

^2^Faculty of Science, University of Basel, Mattenstrasse 26, CH-4058 Basel, Switzerland.

^+^Equal contribution

^*^Corresponding author. E-mail: fussenegger@bsse.ethz.ch

**Contents**

1. **Supplementary Figures 1-14**
2. **Supplementary Tables 1-3**
3. **Supplementary References**

**
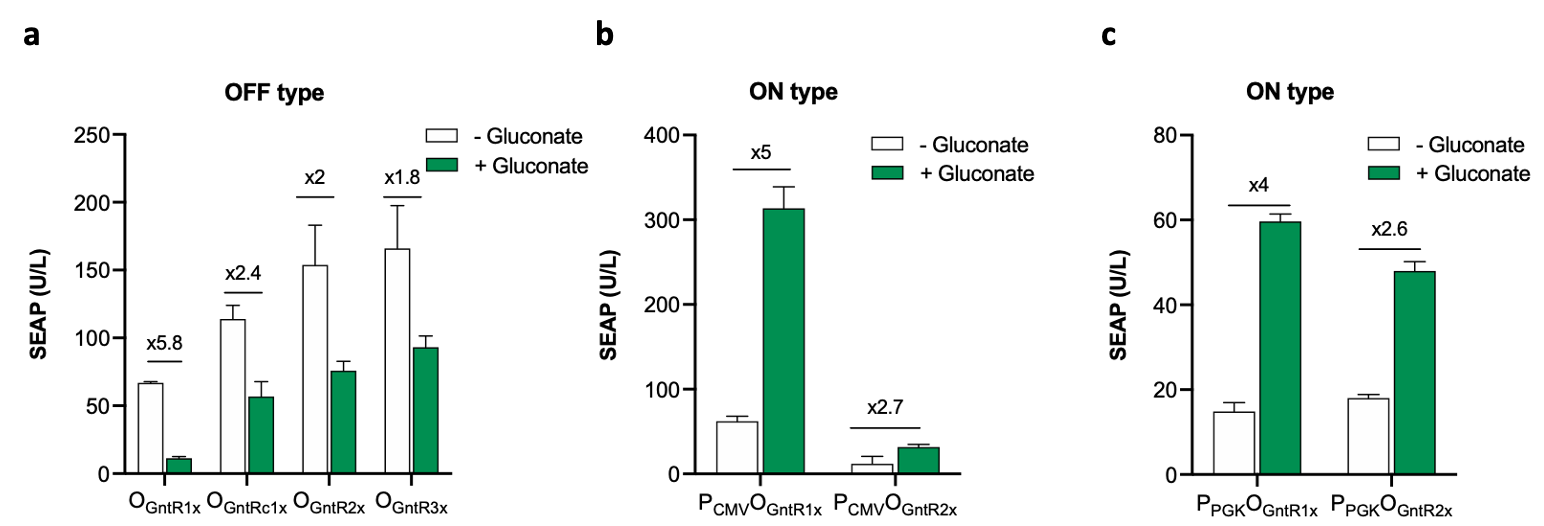
**

**Figure S1. Assessment of promoter variants in the reporter plasmids of the OFF- and ON-type switches.** **a.** SEAP expression in transfected cells constitutively expressing GntR-VPR and reporter constructs with 1×, 2× or 3× O_GntR_ binding sites in the promoter region upstream of the SEAP gene. O_GntR_ is the operator site in *E. coli*’s promoter region of the *gntUK* operon (ATGTTACCGATAACAG) and O_GntRc_ is the consensus sequence (ATGTTACCCGTAACAT) (Porco et al., 1997). **b-c.** SEAP expression in transfected cells constitutively expressing GntR-KRAB and reporter constructs with 1× or 2× O_GntR_ binding sites downstream of the CMV promoter (**b**) or PGK promoter (**c**). Data are shown as mean ± SD of n = 3 biologically independent samples, representative of 3 independent experiments. Numbers above the bars indicate fold difference in SEAP expression level between gluconate-treated and non-treated cultures.

**Figure S2. Effect of mutations in the promoter region of the OFF-type switch.** SEAP expression in cells constitutively expressing GntR-VPR and one of three reporter constructs: with O_GntR_ (ATGTTACCGATAACAG), with O_GntRm_ (ATGTTACTGCGATAACAG) or without any GntR-binding sequence upstream of a minimal promoter (P_min_). Cells were incubated for 24 h in the absence or presence of gluconate (10 mM). Data are shown as mean ± SD of n = 3 biologically independent samples, representative of 3 independent experiments. ns, not significant, **** p < 0.0001.

**
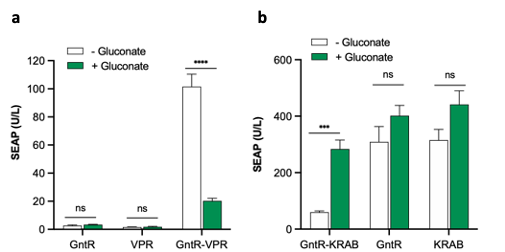
**

**Figure S3. Gene switches with truncated modules are nonfunctional. a.** SEAP expression in cells constitutively expressing GntR (pAna280), VPR (pVH15) or GntR-VPR (pAna274) and the reporter construct with O_GntR_ in the promoter region upstream of the SEAP gene (pAna268). **b.** SEAP expression in cells constitutively expressing GntR-KRAB (pAna283), GntR (pAna280) or KRAB (pAna401) and the reporter construct with the O_GntR_ sequence between the constitutive promoter P_hCMV_ and the SEAP gene (pAna299). Cells were incubated for 24 h in the absence or presence of gluconate (10 mM). Data are shown as mean ± SD of n = 3 biologically independent samples, representative of 3 independent experiments. ns, not significant, *** p < 0.001, **** p < 0.0001.

**Figure S4. The placement of the operator sequence in the promoter regions of the OFF- and ON-type switches influences the switch performance. a.** SEAP expression in cells constitutively expressing GntR-VPR and reporter constructs with the O_GntR_ binding site either upstream (pAna268) or downstream (pAna399) of P_min_ in the regulatory region of the SEAP gene. **b.** SEAP expression in cells constitutively expressing GntR-KRAB and reporter constructs with the O_GntR_ binding site either downstream (pAna299) or upstream (pAna398) of P_hCMV_ in the regulatory region of the SEAP gene. Cells were incubated for 24 h in the absence or presence of gluconate (10 mM). Data are shown as mean ± SD of n = 3 biologically independent samples, representative of 3 independent experiments. ns, not significant, *** p < 0.001, **** p < 0.0001.

**
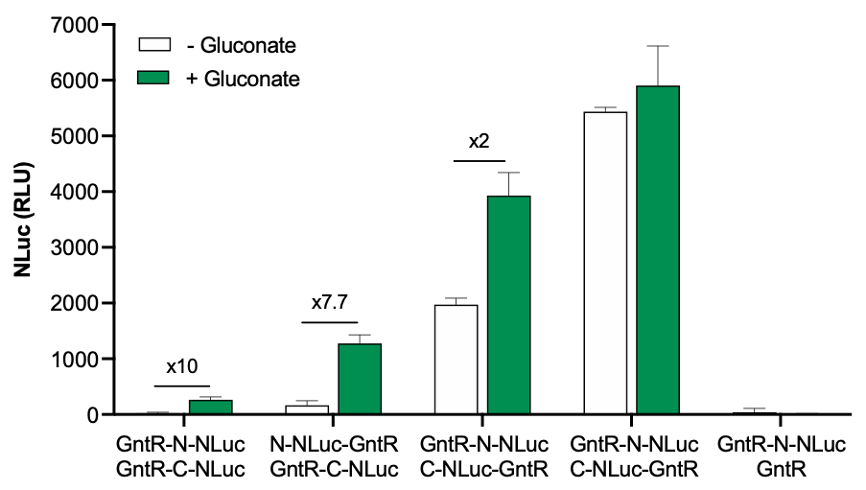
**

**Figure S5. Split NLuc dimerization assay.** Intracellular NLuc luminescence from transiently transfected cells constitutively expressing two GntR fusion constructs, each fused N- or C-terminally to the NLuc split parts as indicated. Cells were cultured for 24 h in the absence or presence of gluconate (10 mM). Data are shown as mean ± SD of n = 3 biologically independent samples, representative of 3 independent experiments. Numbers above the bars indicate fold difference in NLuc expression level between gluconate-treated and non-treated cultures.

**
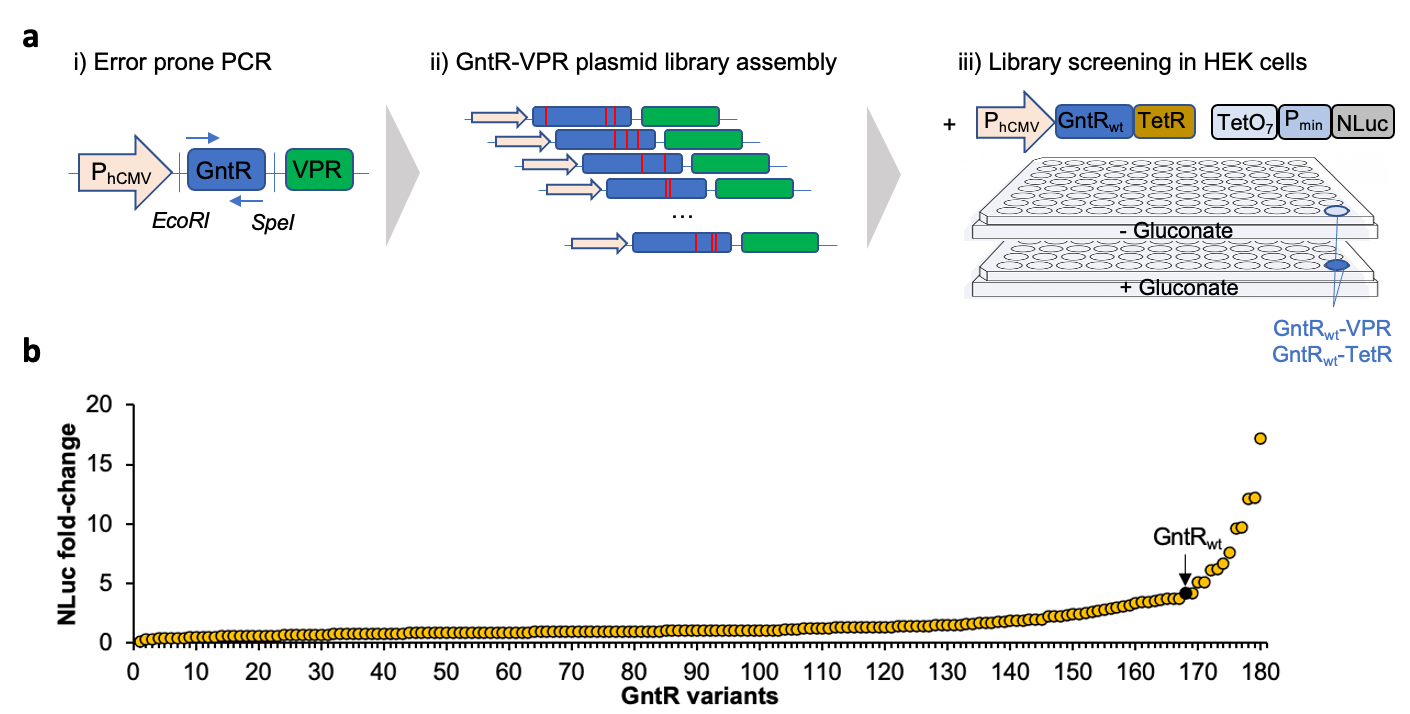
**

**Figure S6. Random mutagenesis of GntR and high-throughput assessment of variants.** **a.** Scheme depicting the steps of the assay for rapid evaluation of the functionality of the mutants. The region between the restriction sites EcoRI/SpeI in the GntR-VPR construct (pAna274) was amplified by error-prone PCR, ligated with the template plasmid (EcoRI/SpeI), and amplified in competent bacteria growing in 96-deep-well plates. A library of 180 plasmid variants was purified and collected in a 96-well format, mixed with pAna323 (P_CMV_-GntR_wt_-TetR) and a TetR-reponsive NLuc reporter plasmid, and used to transfect HEK293T cells seeded in 96-well culture plates. Each variant was used to transfect cells in wells at the same position in two plates, one in gluconate-free medium and the other in gluconate-containing medium. **b.** Primary screening of GntR-VPR plasmid library. Fold-change in NLuc secretion by transfected cells cultured in the absence or presence of gluconate (5 mM). Fold-change obtained with the wild-type GntR is indicated and all variants with higher fold-changes were selected for secondary screening.

**
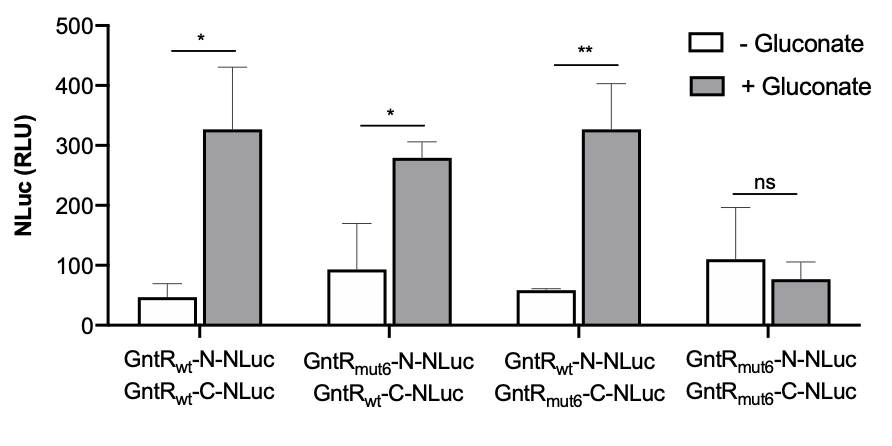
**

**Figure S7. Split NLuc dimerization assay with wild-type and mutant GntR variants.** Intracellular NLuc luminescence from transiently transfected cells constitutively expressing two GntR fusion constructs with NLuc split parts fused to their C-terminus as indicated. Cells were cultured for 24 h in the absence or presence of gluconate (10 mM). Data are shown as mean ± SD of n = 3 biologically independent samples, representative of 3 independent experiments. ns, not significant, * p < 0.05, ** p < 0.01.

**
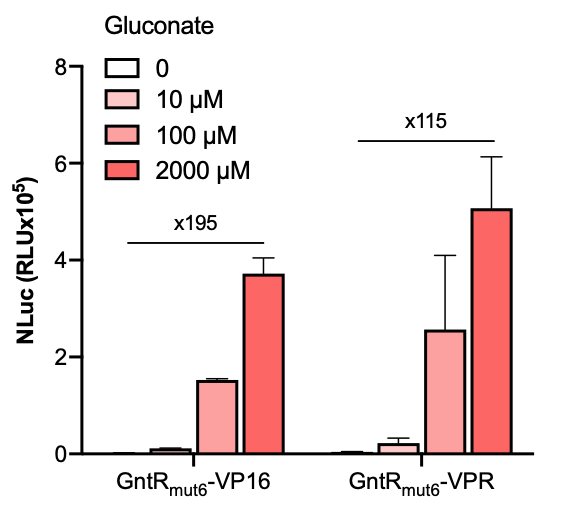
**

**Figure S8**. **Functionality comparison using VPR or VP16 transactivation domains.** HEK293T cells were transfected with pAna323 (P_hCMV_-GntR_wt_-TetR-pA), pTS1017 (TetO_7_-P_min_-NLuc-pA) and either pAna272_mut6_ (P_hCMV_-GntR_mut6_-VP16-pA) or pAna274_mut6_ (P_hCMV_-GntR_mut6_-VPR-pA). Cells were cultivated for 24 h in medium supplemented with different gluconate concentrations. Numbers above the bars indicate fold difference in NLuc expression level between gluconate-treated (2 mM) and non-treated cultures. Data are shown as mean ± SD of n = 3 biologically independent samples, representative of 3 independent experiments. Numbers above the bars indicate fold difference in NLuc expression level between gluconate-treated (2 mM) and non-treated cultures.


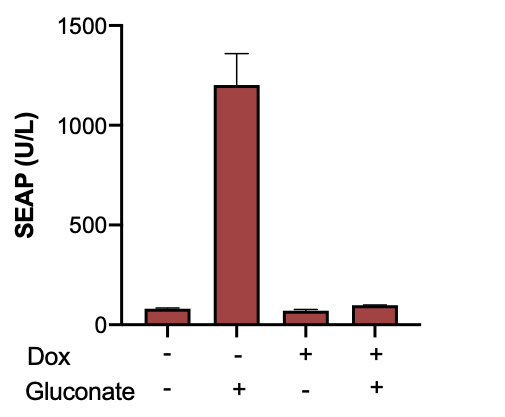


**Figure S9**. **Characterization of N-imply gate genomically integrated.** SEAP production by HEK293T cells constitutively expressing GntR_mut6_-VP16 and GntR-TetR, and encoding SEAP downstream of a tetracycline-responsive promoter. The engineered cells were cultured for 48 h in the presence or absence of Gluconate (20 mM) or Dox (1 μg/ml), as indicated. Data are shown as mean ± SD of n = 3 biologically independent samples, representative of 3 independent experiments.

**Figure S10**. **In vivo control experiments**. Non-functional cell implants failed to increase SEAP levels in the blood of gluconate-treated mice. Functional cell implants produced increased SEAP levels in the blood of gluconate-treated mice, but not mice fed with rice. Non-functional cell implants consist of alginate-encapsulated HEK cells transfected with all of the switch component s except one, namely pAna272-mut6 (P_hCMV_-GntR_R285C/V289I_-VP16-pA). Functional cell implants consist of alginate-encapsulated HEK cells transfected with pAna272-mut6, pAna323 (P_hCMV_-GntR-TetR-pA), pAna302 (P_hCMV_-GntT-pA), and pTS1017 (P_TRE_-SEAP-pA). Blood SEAP levels were measured 24 h after treatment of mice with PBS (- Gluconate), gluconate (5 g/kg) or a rice smoothie (5 g/kg) (equivalent to 375 g of rice for a 75-kg body-weight human) by o.g.. Data are shown as ﻿mean ± SEM of n = 5 mice. Statistical significance was calculated by unpaired t-test. ns, not significant.

**Figure S11**. **In vivo imaging of gluconate-responsive NLuc expression.** **a**. Mice hydrodynamically injected with components for gluconate-switched NLuc production (pSG267, P_hCMV_-GntRmut-VP16-2A-GntR-TetR-pA and pAna345, P_TRE_-NLuc_intra_-pA) were imaged 24 h after gluconate (bottom image) or saline solution (top image) administration (5 g/kg) by o.g.. Luminescence images were recorded by the IVIS imaging system (*n* = 5 mice). **b.** Quantification of the luminescence intensity of images in a.

**
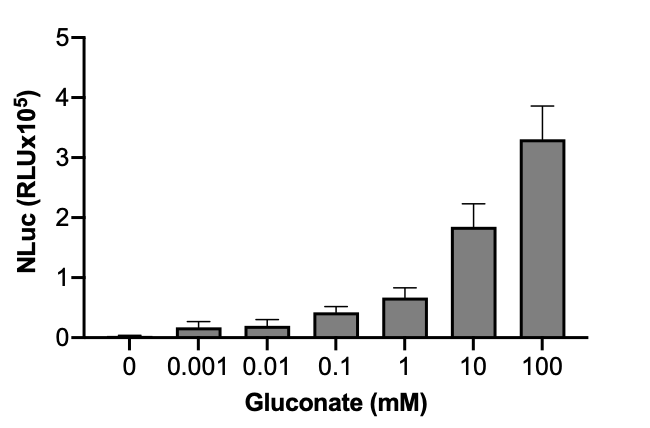
**

**Figure S12**. In vitro validation of vectors encoding gluconate-actuated insulin secretion. HEK293T cells were transfected with pAna366 and pAna368 and cultivated in medium with different gluconate concentrations. After 24 hours, the supernatant was collected and added to an insulin signaling reporter cell line, which consists of HEK cells constitutively expressing the insulin receptor (P_hCMV_-IR-pA) and Elk1-TetR fusion protein, and transiently transfected with a tetracycline-responsive promoter driving NLuc expression (pTS1017, TetO_7_-P_min_-NLuc-pA) (Ye et al., 2016). When insulin binds the insulin receptor, it initiates a signaling cascade that leads to phosphorylation of Elk1-TetR, which can translocate to the nucleus and activate NLuc expression from the TetO_7_ promoter. Data are shown as mean ± SD of n = 4 biologically independent samples.

**Figure S13**. **Mouse body weight and liver enzyme levels during treatment.** **a.** Box plot of mouse body-weight (lines indicate the mean). **b.** Alanine transaminase (ALT) and **c.** Aspartate aminotransferase (AST) activity in the blood of wild-type and T1D mice. T1D mice were hydrodynamically injected with components for gluconate-switched insulin production and then either treated with sodium gluconate or saline solution twice per day by oral gavage. Data are shown as ﻿mean ± SEM of n = 4 mice. Statistical significance was calculated by unpaired t-test. ns, not significant.

**Figure S14**. **Gluconate by itself does not affect diabetic markers.** **a.** Glucose tolerance test (GTT). The GTT was performed by i.p. administration of D-glucose after fasting for 12 h. **b.** Fasting glucose levels of T1D mice either untreated or treated with sodium gluconate twice per day by oral gavage. Data are shown as ﻿mean ± SEM of n = 4 mice. Statistical significance was calculated by unpaired t-test. ns, not significant.

**Table S1. Plasmids used and designed in this study**

| Plasmid name | Plasmid description and cloning details | Source |
| --- | --- | --- |
| pAB300 | Constitutive rTetR expression vector (P_hCMV_-rTetR-pA). | Bertschi et al., 2023 |
| pAB1200 | Reporter vector encoding secreted NLuc under a tetracycline responsive promoter (TetO_7_-P_CMVmin_-ssNluc). | Bertschi unpublished |
| pJH1040 | SEAP-2A-mIns expression driven by a synthetic promoter containing antioxidant response elements (ARE) (P_ARE_-SEAP-2A-mInsulin-pA). | Huang et al., unpublised |
| pMM506 | Constitutive TetR-VP16 expression vector (P_hCMV_-TetR-VP16-pA). | Muller et al., 2017 |
| pTS395 | Constitutive SB100 Sleeping Beauty transposase expression vector (P_hCMV_-SB100-pA). | Haellman et al., 2021 |
| pTS1017 | Reporter vector encoding SEAP under a tetracycline responsive promoter (TetO_7_-P_CMVmin_-SEAP). | Haellman et al., 2021 |
| pTS1018 | Vector encoding P_hCMV_-p2A. | Haellman et al., 2021 |
| pTS1022 | Constitutive SEAP expression vector (P_hCMV_-SEAP-pA). | Haellman et al., 2021 |
| pTS2337 | Sleeping Beauty transposon vector containing two expression cassettes and constitutive expression of mTBFP-2A-Puro^R^ (5’ITR-MCS_1_-pA_1_-MCS_2_-pA_2_-P_RPBSA_-mTBFP-2A-Puro^R^-pA_3_-3’ITR). | Strittmatter unpublised |
| pTS2341 | Sleeping Beauty transposon vector containing two empty expression cassettes and constitutive expression of Puro^R^ (5’ITR-MCS_1_-pA_1_-MCS_2_-pA_2_-P_RPBSA_-Puro^R^-pA_3_-3’ITR). | Strittmatter unpublised |
| pTS2365 | Constitutive VP16 expression vector (P_hCMV_-TetR-pA). | Strittmatter unpublised |
| pTS2367 | Constitutive VP16 expression vector (P_hCMV_-VP16-pA). | Strittmatter unpublised |
| pTS2372 | Constitutive TetR-KRAB expression vector (P_hCMV_-TetR-KRAB-pA). | Strittmatter unpublised |
| pVH15 | Constitutive VPR expression vector (P_hCMV_-VPR-pA). | Haellman  unpublished |
| pVH235 | Constitutive rTetR-VP16 expression vector (P_hCMV_-rTetR-VP16-pA). | Haellman et al., 2021 |
| pVH272 | Constitutive P_PGK_-driven Neo^R^ expression vector (P_PGK_-Neo^R^-pA). | Haellman  unpublished |
| pWP003b | Constitutive VanR-tetR expression vector (P_hCMV_-VanR-TetR-pA). | Bertschi et al., 2023 |
| pAna117 | Constitutive TlpA39-VP16 expression vector (P_hCMV_-TlpA-VP16-pA). | Stefanov et al., 2021 |
| pAna148 | Constitutive TlpA39-KRAB expression vector (P_hCMV_-TlpA-KRAB-pA). KRAB was PCR-amplified from pTS2372 using oligos oAPT156 and oAPT157. The PCR product was digested with BsiWI/XbaI and ligated into pAna117 (BsiWI/XbaI). | This work |
| pAna157 | Constitutive P_PGK_-driven Puro^R^ expression vector. Puro^R^ was PCR-amplified from BB6-RPBSA-YPet-2A-Puro^R^ with oligos oAPT162 and oAPT163. The PCR product was digested with EcoRI/HindIII and ligated into pVH272 (EcoRI/HindIII). | This work |
| pAna268 | Reporter vector encoding O_GntR_P_min_-driven SEAP expression (O_GntR_P_min_-SEAP-pA). O_GntR_P_min_ was assembled by phosphorylating and annealing oligos oAPT263 and oAPT264. The annealed oligos were ligated into pTS1022 (XhoI/EcoRI). | This work |
| pAna268b | Reporter vector encoding O_GntRc1x_P_min_-driven SEAP expression (O_GntRc1x_P_min_-SEAP-pA). O_GntRc1x_P_min_ was assembled by phosphorylating and annealing oligos oAPT341 and oAPT342. The annealed oligos were ligated into pTS1022 (XhoI/EcoRI). | This work |
| pAna272 | Constitutive GntR-VP16 expression vector (P_hCMV_-GntR-VP16-pA). GntR was synthesized by Twist Biosciences, digested using EcoRI/NheI and ligated into pTS2367 (EcoRI/SpeI). | This work |
| pAna272-mut6 | Constitutive GntR_R285C/V289I_-VP16 expression vector (P_hCMV_-GntR_R285C/V289I_-VP16-pA). GntR_R285C/V289I_ was excised from pAna274mut1 with EcoRI/SpeI and ligated into pTS2367 (EcoRI/SpeI). | This work |
| pAna274 | Constitutive GntR-VPR expression vector (P_hCMV_-GntR-VPR-pA). GntR was synthesized by Twist Biosciences, digested using EcoRI/SpeI and ligated into pVH15 (EcoRI/SpeI). | This work |
| pAna274-mutants | Constitutive GntRmut-VPR expression vector (P_hCMV_-GntR-VPR-pA). A library of GntR variants (GntRmut) was error-prone PCR-amplified from pAna274 using oligos oAPT309 and oAPT310. The PCR product was digested using EcoRI/SpeI and ligated into pVH15 (EcoRI/SpeI). | This work |
| pAna278 | Reporter vector encoding O_GntR2x_P_min_-driven SEAP expression (O_GntR2x_P_min_-SEAP-pA). O_GntR2x_P_min_ was assembled by phosphorylating and annealing oligos oAPT269 and oAPT270. The annealed oligos were ligated into pAna268 (XhoI/PacI). | This work |
| pAna279 | Reporter vector encoding O_GntR3x_P_min_-driven SEAP expression (O_GntR3x_P_min_-SEAP-pA). O_GntR3x_P_min_ was assembled by phosphorylating and annealing oligos oAPT271 and oAPT272. The annealed oligos were ligated into pAna268 (XhoI/PacI). | This work |
| pAna280 | Constitutive GntR expression vector (P_hCMV_-GntR-pA). GntR was synthesized by Twist Biosciences, digested using EcoRI/NheI and ligated into pTS1022 (EcoRI/NheI). | This work |
| pAna283 | Constitutive GntR-KRAB expression vector (P_hCMV_-GntR-KRAB-pA). KRAB was PCR-amplified from pAna148 using oligos oAPT277 and oAPT278. The PCR product was digested with NheI/BamHI and ligated into pAna280 (NheI/BamHI). | This work |
| pAna299 | Reporter vector encoding P_hCMV_O_GntR_-driven SEAP expression (P_hCMV_O_GntR_ -SEAP-pA). O_GntR_ was assembled by phosphorylating and annealing oligos oAPT287 and oAPT288. The annealed oligos were ligated into pTS1022 (EcoRI/SpeI). | This work |
| pAna300 | Reporter vector encoding P_hCMV_O_GntR2x_-driven SEAP expression (P_hCMV_O_GntR2x_-SEAP-pA). O_GntR2x_ was assembled by phosphorylating and annealing oligos oAPT289 and oAPT290. The annealed oligos were ligated into pTS1022 (EcoRI/SpeI). | This work |
| pAna302 | Constitutive GntT expression vector (P_hCMV_-GntT-pA). GntT was synthesized by Twist Biosciences, digested with EcoRI/NheI and ligated into pTS1022 (EcoRI/NheI). | This work |
| pAna322 | Constitutive TetR-GntR expression vector (P_hCMV_-TetR-GntR-pA). TetR was PCR-amplified from pMM506 (P_hCMV_-TetR-VP16-pA) using oligos oAPT306 and oAPT307. The PCR product was digested with EcoRI/NheI and ligated into pAna280 (EcoRI/SpeI). | This work |
| pAna323 | Constitutive GntR-TetR expression vector (P_hCMV_-GntR-TetR-pA). TetR was PCR-amplified from pWP003b using oligos oAPT308 and oAPT157. The PCR product was digested with SpeI/BamHI and ligated into pAna280 (NheI/BamHI). | This work |
| pAna323-mut6 | Constitutive GntR_R285C/V289I_-TetR expression vector (P_hCMV_-GntR_R285C/V289I_-TetR-pA). pAna323 was PCR-amplified using oligos oAPT311 and oAPT312 to insert a SpeI site upstream of TetR. The PCR product was digested with EcoR/SpeI and ligated with GntR_R285C/V289I_ excised from pAna274mut1 (EcoRI/SpeI). | This work |
| pAna324 | Constitutive GntR-rTetR-GntR expression vector (P_hCMV_-GntR-rTetR-pA). GntR was excised from pAna280 with EcoRI/NheI and ligated into pAB300 (P_hCMV_-rTetR-pA) (EcoRI/SpeI). | This work |
| pAna324-mut6 | Constitutive GntR_R285C/V289I_-rTetR expression vector (P_hCMV_-GntR_R285C/V289I_-rTetR-pA). GntR_R285C/V289I_ was excised from pAna274-mut1 with EcoRI/SpeI and ligated into pAB300 (EcoRI/SpeI). | This work |
| pAna325 | Reporter vector encoding P_hCMV_O_GntR_-driven secreted NLuc expression (O_GntR_P_min_-ssNLuc-pA). Secreted NLuc was excised from pAB1200 with SpeI/AgeI and ligated into pAna299 (SpeI/AgeI). | This work |
| pAna333 | Reporter vector encoding P_min_-driven SEAP expression (P_min_-SEAP-pA). P_min_ was assembled by phosphorylating and annealing oligos oAPT319 and oAPT320. The annealed oligos were ligated into pAna268 (XhoI/PacI). | This work |
| pAna342 | Constitutive GntR-TetR expression vector (P_hCMV_-GntR_R285C/V289I_-TetR-p2A-MCS-pA). GntR-TetR was excised from pAna323 with EcoRI/NheI and ligated into pTS1018 (EcoRI/SpeI). | This work |
| pAna343 | Constitutive GntR-TetR-2A-GntT expression vector (P_hCMV_-GntR-TetR-p2A-GntT-pA). GntR was PCR-amplified from pAna302 with oligos oAPT324 and oAPT325. The PCR product was digested with NheI/HindIII and ligated into pAna342 (NheI/HindIII). | This work |
| pAna345 | Reporter vector encoding NLuc under a tetracycline responsive promoter (P_TRE_-Nluc_intra_-pA). NLuc was PCR-amplified from pAB1200 using oligos oAPT202 and oAPT205. The PCR product was digested with SpeI/BamHI, and ligated into pTS1017 (SpeI/BamHI). | This work |
| pAna346 | Constitutive GntR_R285C/V289I_-VP16-2A expression vector (P_hCMV_- GntR_R285C/V289I_-VP16-2A-MCS-pA). GntR_R285C/V289I_-VP16 was excised from pAna272mut6 with EcoRI/NheI and ligated into pTS1018 (EcoRI/SpeI). | This work |
| pAna366 | Sleeping Beauty transposon vector containing three expression cassettes: one encoding P_hCMV_-driven GntR_mut6_-VP16, the second encoding SEAP-2A-mIns under a tetracycline responsive promoter and the third encoding P_RPBSA_-driven mTBFP-2A-Puro^R^ (5’ITR-MCS_1_-pA_1_-TetO_7_P_min_-SEAP-2A-mIns-pA_2_-P_RPBSA_-mTBFP-2A-Puro^R^-pA_3_-3’ITR). P_hCMV_-GntR_mut6_-VP16 was excised from pAna272_mut6_ (MluI/HindIII) and ligated into pAna354 (MluI/HindIII). | This work |
| pAna368 | Sleeping Beauty transposon vector containing two expression cassettes, one encoding P_hCMV_-driven GntR-TetR-p2A-GntT and the second encoding constitutive expression of Puro^R^ (5’ITR-MCS_1_-pA_1_-P_hCMV_-GntR-TetR-p2A-GntT-pA_2_-P_RPBSA_-Puro^R^-pA_3_-3’ITR). P_hCMV_-GntR-TetR-p2A-GntT-pA was excised from pAna343 (MluI/HindIII) and ligated into pTS2342 (BsaI). | This work |
| pAna398 | Reporter vector encoding O_GntR_P_hCMV_-driven SEAP expression (O_GntR_P_hCMV_-SEAP-pA). O_GntR_ was assembled by phosphorylating and annealing oligos oAPT365 and oAPT366. The annealed oligos were ligated into pTS1022 (MluI/XhoI). | This work |
| pAna399 | Reporter vector encoding P_min_O_GntR_-driven SEAP expression (P_min_O_GntR_-SEAP-pA). P_min_O_GntR_ was assembled by phosphorylating and annealing oligos oAPT367 and oAPT368. The annealed oligos were ligated into pTS1022 (MluI/EcoRI). | This work |
| pAna400 | Reporter vector encoding O_GntRm_P_min_-driven SEAP expression (O_GntRm_P_min_-SEAP-pA). O_GntRm_ was assembled by phosphorylating and annealing oligos oAPT369 and oAPT370. The annealed oligos were ligated into pAna268 (MluI/PacI). | This work |
| pAna401 | Constitutive KRAB expression vector (P_hCMV_-KRAB-pA). pAna283 was digested with SpeI/NheI to cut GntR out and re-ligated. | This work |
| pSG267 | Constitutive GntR_R285C/V289I_-VP16-2A-GntR-TetR expression vector (P_hCMV_-GntR_R285C/V289I_-VP16-2A-GntR-TetR-pA). GntR-TetR was excised from pAna323 using SpeI/HindIII and ligated into pAna346 (NheI/HindII). | This work |

**Abbreviations and additional information: 5’/3’ITR**, 5’/3’inverted terminal repeat of Sleeping Beauty transposase; **GntR**, gluconate-responsive transrepressor from *E. coli*; **KRAB**, Kruppel-associated box domain; **MCS**, multiple cloning site; **mTagBFP**, blue fluorescent protein derived from *Entacmaea quadricolor*; **mIns**, mouse insulin; **NLuc**, nanoluciferase; **O_GntRn_**, GntR-specific operator with n number of operator sites; **p2A**, self-cleaving peptide; **pA**, polyadenylation signal; **P_min_**, weak pGL4.23-derived minimal promoter; **P_hCMV_**, human cytomegalovirus immediate-early promoter; **P_hCMVmin_**, minimal version of P_hCMV_; **P_PGK_**, mouse phosphoglycerate kinase 1 promoter; **P_RPBSA_**, synthetic promoter made up of a fragment of the RPL13a promoter fused to a region of the RPL41 gene; **Puro^R^**, puromycin resistance gene puromycin N-acetyl-transferase; **rTetR**, reverse tetracycline-dependent transcription factor; **SEAP**, human placental secreted alkaline phosphatase; **TetO_7_**, TetR-specific operator with 7 operator sites; **TetR**, tetracycline-dependent transcription factor; **VP16**, herpes simplex virus-derived transactivation domain; **VPR**, tripartite mammalian transactivator VP64-p65-Rta.

**Table S2. Oligonucleotide sequences used in this work**

| **Oligo ID** | **Oligonucleotide Sequence (5’-3’)** |
| --- | --- |
| oAPT156 | TAAGCGTACGGGTGGTTCTGGTCCAGATC |
| oAPT157 | TAAGTCTAGACACCGGTGGATCC |
| oATP162 | TAAGGAATTCACCATGACTAGTACCGAGTACAAGCCCACGGTGC |
| oATP163 | TAAGAAGCTTCTAGGCACCGGGCTTGCGGG |
| oATP263 | TCGAGATGTTACCGATAACAGTTAATTAATAGAGGGTATATAATGGAAGCTCGACTTCCAGG |
| oATP264 | AATTCCTGGAAGTCGAGCTTCCATTATATACCCTCTATTAATTAACTGTTATCGGTAACATC |
| oATP271 | TCGAGATGTTACCGATAACAGTAGGCCCGCGAGATGTTACCGATAACAGGGTACCAGCGCTATGTTACCGATAACAGTTAAT |
| oATP272 | TAACTGTTATCGGTAACATAGCGCTGGTACCCTGTTATCGGTAACATCTCGCGGGCCTACTGTTATCGGTAACATC |
| oATP277 | TAAGGCTAGCAGCCGCGCGCCAGATCCAAAAAAGAAGAGAAA |
| oATP278 | TAAGGGATCCCCAGAGATCATTCCTTGCC |
| oATP287 | AATTCATGTTACCGATAACAGACCATGA |
| oATP288 | CTAGTCATGGTCTGTTATCGGTAACATG |
| oATP289 | AATTCATGTTACCGATAACAGAGTAAACTCTTCATACGTTATGTTACCGATAACAGACCATGA |
| oATP290 | CTAGTCATGGTCTGTTATCGGTAACATAACGTATGAAGAGTTTACTCTGTTATCGGTAACATG |
| oATP306 | AGCGGAATTCACCATGACTAGT |
| oATP307 | TAAGGCTAGCGGACCCACTTTCACATTTAAGTT |
| oATP308 | TAAGACTAGTGGTGGGGGGTCTGCTAGT |
| oATP309 | AGCGGAATTCACCATGAAAAAAAAGCGACCTGTGCTGC |
| oATP310 | TAAGACTAGTGATGCTCCCCCCAGGGCTA |
| oATP311 | TAAGACTAGTGGTGGGGGGTCTG |
| oATP312 | CATGGTGAATTCCGCTTCGA |
| oAPT319 | TCGAGCATCTATCATCTAAAAAACCAGAAAAACAAATAACATCATGTTTTTAAACTAATTAAATGAAATAAAATTTTAAGCCACTCGCCATTAAT |
| oAPT320 | TAATGGCGAGTGGCTTAAAATTTTATTTCATTTAATTAGTTTAAAAACATGATGTTATTTGTTTTTCTGGTTTTTTAGATGATAGATGC |
| oATP324 | TAAGGCTAGCCCCCTCGTCATCGTGGCC |
| oATP325 | TAGGAAGCTTTCTAGAcACCGGTGGATCCGATCACCATATTCAGAAGCAGACAGC |
| oATP341 | TCGAGATGTTACCCGTAACATTTAATTAATAGAGGGTATATAATGGAAGCTCGACTTCCAGG |
| oATP342 | AATTCCTGGAAGTCGAGCTTCCATTATATACCCTCTATTAATTAAATGTTACGGGTAACATC |
| oAPT365 | CGCGTATGTTACCGATAACAGC |
| oAPT366 | TCGAGCTGTTATCGGTAACATA |
| oAPT367 | CGCGTTAGAGGGTATATAATGGAAGCTCGACTTCCAGTTAATTAAATGTTACCGATAACAGG |
| oAPT368 | AATTCCTGTTATCGGTAACATTTAATTAACTGGAAGTCGAGCTTCCATTATATACCCTCTAA |
| oAPT369 | CGCGTGGTACCCTCGAGATGTTACTGCGATAACAGTTAAT |
| oAPT370 | TAACTGTTATCGCAGTAACATCTCGAGGGTACCA |

**Table S3. Mutations of the six GntR variants confirmed in the secondary screening.**

| **Mutant nr.** | **Mutated amino acids** |
| --- | --- |
| mut1 | GntR_G92R_ |
| mut2 | GntR_D12V/S58T/K198R_ |
| mut3 | GntR_V210L/E227G_ |
| mut4 | GntR_D55V/E235K/G279V_ |
| mut5 | GntR_A306S_ |
| mut6 | GntR_R285C/V289I_ |

**GntR sequence**

ATGAAAAAAAAGCGACCTGTGCTGCAGGACGTTGCCGATAGGGTTGGAGTCACAAAGATGACCGTAAGTAGATTCCTTAGAAATCCCGAGCAGGTTTCAGTGGCACTTCGGGGTAAGATTGCCGCCGCCCTAGATGAGCTGGGCTACATCCCTAACCGGGCACCTGACATCCTGTCCAACGCAACCAGTCGCGCTATTGGAGTGCTTCTCCCAAGCCTGACTAACCAGGTTTTTGCCGAGGTGCTAAGAGGAATCGAGAGCGTGACAGATGCGCATGGGTATCAAACTATGCTGGCTCATTACGGGTATAAACCTGAGATGGAGCAGGAGAGGCTCGAAAGCATGTTAAGTTGGAATATTGATGGCTTGATCCTCACAGAGAGGACTCACACCCCCAGGACGCTGAAGATGATCGAAGTAGCAGGTATACCAGTCGTCGAGTTGATGGACTCAAAATCCCCATGCTTAGATATAGCCGTGGGATTCGACAATTTTGAAGCTGCTCGCCAGATGACCACGGCCATTATCGCCCGCGGCCATCGGCACATTGCTTACCTCGGCGCTAGACTGGATGAGCGCACAATAATTAAGCAGAAGGGGTATGAACAGGCAATGCTGGACGCAGGACTGGTGCCCTATTCGGTGATGGTCGAACAGTCTTCTTCTTACTCCAGTGGTATCGAACTCATTAGACAGGCGCGAAGGGAATACCCGCAATTGGACGGCGTGTTTTGCACCAACGACGATTTAGCAGTGGGCGCGGCTTTCGAATGTCAACGGCTGGGCCTGAAGGTGCCCGACGATATGGCCATTGCTGGTTTCCACGGACACGACATCGGGCAAGTCATGGAGCCGCGTCTGGCCTCAGTACTCACTCCCCGGGAGCGCATGGGATCTATCGGCGCCGAGCGTTTGCTGGCCAGAATCCGAGGGGAATCCGTCACCCCAAAAATGCTCGACCTGGGCTTTACACTTAGCCCTGGGGGGAGCATCTAG

**Supplementary References**

Bertschi A, Li P, Galvan S, Teixeira AP, Fussenegger M. Combinatorial protein dimerization enables precise multi-input synthetic computations in human cells. *Nature Chemical Biology* in press

Haellman V, Strittmatter T, Bertschi A, Stücheli P, Fussenegger M, 2021. A versatile plasmid architecture for mammalian synthetic biology (VAMSyB). *Metabolic Engineering*, *66*, pp.41-50.

Muller M, Auslander S, Spinnler A, Auslander D, Sikorski J, Folcher M, Fussenegger M. (2017). Designed cell consortia as fragrance-programmable analog-to-digital converters. Nat Chem Biol *13*, 309-316.

Ye H, Xie M, Xue S, Hamri GCE, Yin J, Zulewski H, Fussenegger M, 2016. Self-adjusting synthetic gene circuit for correcting insulin resistance. *Nature Biomedical Engineering*, *1*(1), p.0005.
